# Transition of tumor-associated macrophages from MHC class II^hi ^to MHC class II^low ^mediates tumor progression in mice

**DOI:** 10.1186/1471-2172-12-43

**Published:** 2011-08-04

**Authors:** Benfan Wang, Qinyan Li, Li Qin, Siting Zhao, Jinyan Wang, Xiaoping Chen

**Affiliations:** 1 Laboratory of Pathogen Biology, State Key Laboratory of Respiratory Disease, Center for Infection and Immunity, Guangzhou Institutes of Biomedicine and Health, Chinese Academy of Sciences, Guangzhou, 510530, China; 2 Department of Medical Biotechnology, School of Life Science, University of Science and Technology of China, Hefei, 230026, China; 3 School of Integrated Traditional Chinese & Western Medicine, Anhui University of Traditional Chinese Medicine, Hefei, 230038, China; 4 Department of Immunology, Institute of Pathology, College of Basic Medical Science, China Medical University, Shenyang, 110001, China

## Abstract

**Background:**

Tumor-associated macrophages (TAMs) are the most abundant immune cells within the tumor stroma and play a crucial role in tumor development. Although clinical investigations indicate that high levels of macrophage (MΦ) infiltration into tumors are associated with a poor prognosis, the exact role played by TAMs during tumor development remains unclear. The present study aimed to investigate dynamic changes in TAM major histocompatibility complex (MHC) class II expression levels and to assess the effects of these changes on tumor progression.

**Results:**

Significant inhibition of tumor growth in the murine hepatocellular carcinoma Hepa1-6 model was closely associated with partial TAM depletion. Strikingly, two distinct TAM subsets were found to coexist within the tumor microenvironment during Hepa1-6 tumor development. An MHC class II^hi ^TAM population appeared during the early phase of tumor development and was associated with tumor suppression; however, an MHC class II^low ^TAM population became increasingly predominant as the tumor progressed.

**Conclusions:**

Tumor progression was positively correlated with increasing infiltration of the tumor tissues by MHC class II^low ^TAMs. Thus, targeting the transition of MΦ may be a novel strategy for drug development and immunotherapy.

## Background

Macrophages (MΦ) represent the most abundant immune cell population in the tumor microenvironment and play a key role in tumor development [1,2]. High levels of MΦ infiltration into tumor tissues are associated with a poor prognosis; this is particularly true for hepatocellular carcinoma (HCC) [3-6]. Although a decreased number of macrophages correlates with a reduction in tumor growth in several tumor graft models [7,8], there are some exceptions. For example, depletion of Kupffer cells worsens the prognosis of tumor-bearing mice in peritoneal xenograft models because the cancer cells are able to metastasize to the liver; thus, the mice die from the increased tumor burden in the absence of MΦ [9,10]. These contradictory reports highlight the fact that little is known about the exact role of tumor-associated macrophages (TAMs) during tumor development.

MΦ are a highly heterogeneous cell population. This is because their phenotypes and diverse functions are shaped by the tumor microenvironment [11]. MΦ can be classified on the basis of two distinct activation states. Classically activated MΦ (M1), induced by IFN-γ or microbial products, produce high levels of proinflammatory cytokines (IL-12 and IL-23), express major histocompatibility complex (MHC) molecules and iNOS, and act as the primary source of anti-tumor immune cells [12-14]. In contrast, alternatively activated MΦ (M2), polarized by IL-4 or/and IL-13, secrete anti-inflammatory cytokines and are characterized by increased arginase-1 activity and the expression of Ym-1, MGL, Fizz1, and MSF [15-17]. Functionally, M2 MΦ are thought to suppress inflammation and to facilitate wound healing by promoting angiogenesis and tissue remodeling [15,18]. A recent study shows that mouse mammary tumors contain phenotypically and functionally distinct TAM subsets, and that these subsets promote tumor growth via different mechanisms [19]. HLA-DR^high ^and HLA-DR^low ^IL-10^+ ^monocytes/MΦ have also been identified in different regions within tumor tissues, where they mediate T cell anergy through PD-L1 and increase tumor cell migration and invasion [3-5,20]. However, the precise role played by these heterogeneous TAM subsets in tumor progression has rarely been reported.

Clodronate (Cl_2_MDP)-encapsulated liposomes cause irreversible damage to MΦ *in vivo*, thereby efficiently decreasing the number of infiltrating MΦ [21,22]. Strategies incorporating liposome-mediated MΦ depletion have been successfully used in murine tumor models of teratocarcinoma, rhabdomyosarcoma, lung cancer, and melanoma [7,23,24].

In the present study, Cl_2_MDP-liposomes were used to partially deplete TAMs in a murine transplanted hepatoma model to investigate the exact role played by TAMs during tumor development and the mechanisms underlying TAM-mediated tumor progression.

## Results

### Partial depletion of MΦ using Cl_2_MDP-liposomes

**Figure 1:**
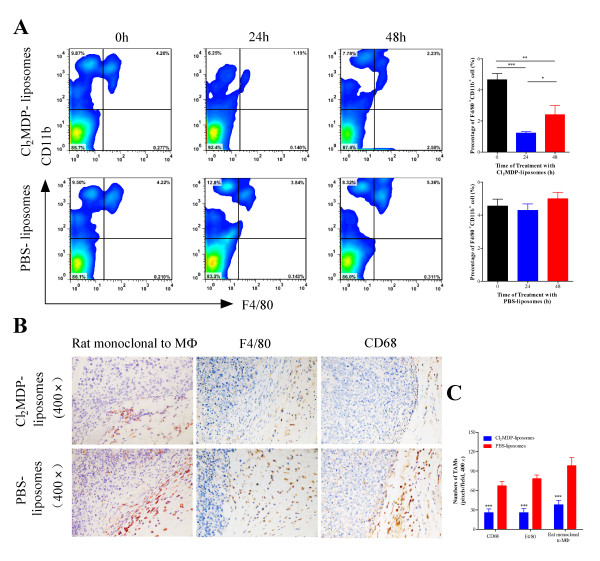
**Effect of Cl_2_MDP-liposome treatment on the survival of MΦ in mice**. (A) Cl_2_MDP-liposome-mediated depletion of monocytes/MΦ from the peripheral blood of mice. Each value represents the mean proportion of monocytes/MΦ ± SD (*n *= 6). (B) The survival of TAMs in tumor-bearing mice was examined by immunostaining with the rat monoclonal anti-MΦ antibody [RM0029-11H3] and antibodies to F4/80 and CD68 after treatment with Cl_2_MDP-liposomes. (C) Infiltrating TAMs were counted at × 400 magnification (a 0.17 mm^2 ^field). Data represent the mean MΦ number per field ± SD (10 fields per tumor, 5 tumors per group). *p < 0.05, **p < 0.01, ***p < 0.001 (unpaired Student's t-test).

To examine the extent of *in vivo *monocyte/MΦ depletion by Cl_2_MDP, naïve mice were intravenously (i.v.) injected with Cl_2_MDP-encapsulating liposomes 2 days before tumor inoculation. As shown in Figure 1A, the proportion of monocytes/MΦ in the peripheral blood was approximately 5-7% before Cl_2_MDP-liposome administration. This proportion decreased markedly (to around 1%, *p *< 0.001) 24 h after the injection of Cl_2_MDP-liposomes, before recovering (to about 3%, *p *< 0.05) 48 h later. The PBS liposome control did not affect the number of monocytes/MΦ *in vivo*. We next examined the *in vivo *effects of Cl_2_MDP-liposomes on TAM survival on Day 20 post-tumor inoculation. Immunohistochemical staining with a rat anti-MΦ monoclonal antibody [RM0029-11H3], anti-F4/80, and anti-CD68 showed that the number of MΦ in tumor tissues was significantly lower than that in control tissues (*p *< 0.001, Figure 1B and 1C). This suggests that Cl_2_MDP-liposomes efficiently deplete the number of TAMs in tumor-bearing mice. In addition, we also assessed the survival of tumor cells treated with Cl_2_MDP-liposomes *in vitro*. The results showed that survival was not significantly affected (data not shown). Thus, it appears that Cl_2_MDP-liposome treatment may partially deplete MΦ without directly affecting the growth of tumor cells *in vivo*.

### Partial depletion of MΦ inhibits tumor progression in tumor-bearing mice

**Figure 2:**
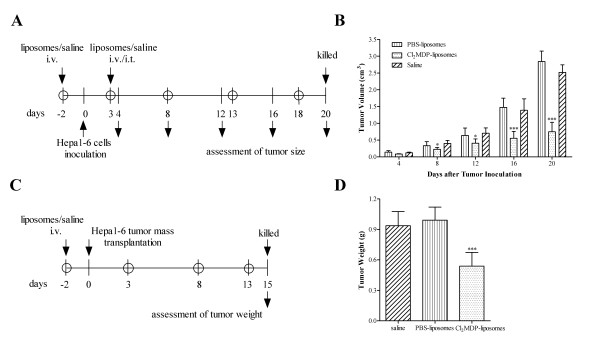
**MΦ depletion by Cl_2_MDP-liposomes inhibits tumor growth in the Hepa1-6 induced tumor model**. (A) Experimental outline for MΦ depletion by administration of Cl_2_MDP-liposomes in s.c. tumor-bearing mice. Saline and PBS-liposomes were used as the controls. (B) Size of the s.c.-transplanted tumors (*n *= 15). Data are representative of three independent experiments. (C) Experimental outline for administration of Cl_2_MDP-liposomes in orthotopic tumor-bearing mice. (D) Weights of orthotopically transplanted tumors (*n *= 12). The bars represent the mean ± SD; *p < 0.05, ***p < 0.001 (unpaired Student's *t*-test).

To determine the effect of TAMs on tumor growth in Hepa1-6 tumor-bearing mice, MΦ were depleted using Cl_2_MDP-liposomes (Figure 2A). As shown in Figure 2B, the rate of subcutaneous (s.c.) tumor growth in the Cl_2_MDP-liposome-treated group was significantly slower than that in the controls (p < 0.001). A similar experiment was also conducted in a murine orthotopic tumor model, in which tumors were induced by implanting small tumor masses into the mouse livers (Figure 2C). The results showed that macrophage depletion mediated by Cl_2_MDP-liposome treatment partially inhibited tumor growth (p < 0.001, Figure 2D). Taken together, these results suggest that Cl_2_MDP-liposome-mediated inhibition of tumor growth is positively correlated with a decrease in TAM infiltration.

### Tumor progression correlates with the level of TAM infiltration

**Figure 3:**
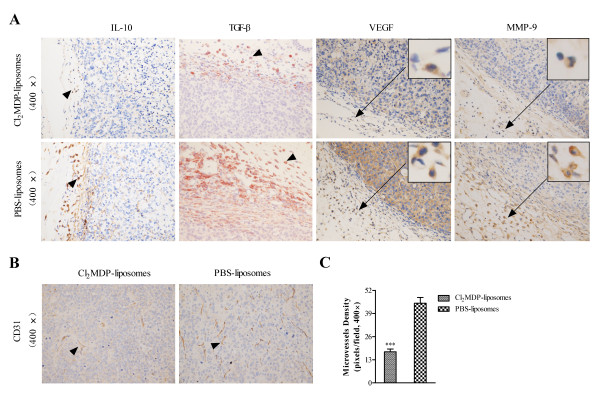
**Immunohistochemical analyses of immunosuppressive molecules after Cl_2_MDP-liposome treatment**. (A) Expression of IL-10, TGF-β, VEGF, and MMP-9 was evaluated by immunostaining. Original magnification: × 400. Insets: representative cells expressing VEGF and MMP-9. Arrowheads show positively-stained cells. (B) Examination of CD31 expression in tumors by immunostaining. (C) CD31-positive microvessel density was evaluated by counting at × 400 magnification (a 0.17 mm^2 ^field). Data are expressed as mean vessel number per field ± SD (10 fields per tumor, 5 tumors per group). ***p < 0.001 (unpaired Student's t-test).

To further examine the role of TAMs in tumor progression in murine models, several TAM-associated suppressive factors were analyzed *in situ*. Figure 3A shows that expression of IL-10, TGF-β, MMP-9 and VEGF in the tumor microenvironment was markedly reduced in the Cl_2_MDP liposome-treated group compared with that in the control vehicle-treated group. This result suggests that increased TAM infiltration contributes to the formation of a suppressive tumor microenvironment and the promotion of tumor growth. This conclusion was verified by examining CD31 expression (Figure 3B,C). Taken together, these data show that tumor infiltration by TAMs plays an important role in tumor progression.

### TAMs in tumor-bearing mice are a heterogeneous population

**Figure 4:**
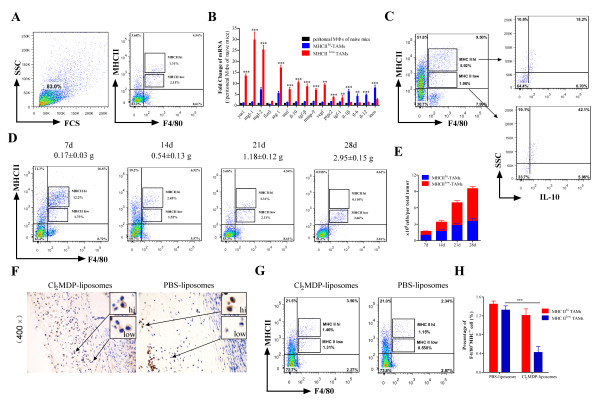
**Infiltration of Hepa1-6 tumors by distinct TAM subsets**. (A) Identification of distinct TAM subsets in single-cell suspensions obtained from tumors on Day 21 after tumor inoculation (*n *= 5). (B) Phenotypic analysis of classically versus alternatively activated TAM subsets using qRT-PCR. Data are representative of three independent experiments. The bars represent the mean ± SD. (C) Expression of IL-10 by sorted TAMs in tumors on Day 10 after tumor inoculation (*n *= 5). (D) Dynamic analysis of two distinct subsets from tumors on Days 7, 14, 21, and 28 after tumor inoculation. Tumor weights are shown above the FACS plots (*n *= 5). (E) Number of TAMs (F4/80^+^MHC II^low ^cell population versus F4/80^+^MHC II^hi ^cell population) per tumor at the indicated time points. (F) Expression of MHC class II by untreated and TAM- depleted tumors analyzed by immunohistochemistry. Insets: representative cells expressing high and low levels of MHC class II (*n *= 5) (× 400 magnification). (G) Analysis of MHC class II^hi ^and MHC class II^low ^TAMs in untreated and TAM-depleted tumors on Day 20 after treatment with Cl_2_MDP-liposomes (*n *= 5). (H) Proportion of MHC class II^hi ^and MHC class II^low ^TAMs in the untreated and TAM-depleted tumors (*n *= 5). The bars represent the mean ± SD; **p <0.01, ***p < 0.001 (unpaired Student's *t*-test).

To further elucidate the mechanisms underlying TAM-mediated tumor growth, TAMs were isolated and phenotypically characterized. Single cells were recovered from tumor tissues at the indicated time points. Two heterogeneous TAM populations were identified based on their expression of MHC class II molecules, namely MHC class II^low ^and MHC class II^hi ^(Figure 4A). Strikingly, the MHC class II^low ^TAM subset expressed high levels of *ym1, mgl1/2, fizz1, arg-1, msf, il-10, tgf-β, mmp-9, vegf*, and *ptge2*, whereas the MHC class II^hi ^TAM subset expressed high levels of *il-1β, il-6, il-12*, and *inos *(Figure 4B). Next, the differential expression of IL-10 between both TAM populations was examined in co-staining experiments. The proportion of IL-10-expressing MΦ in the MHC class II^low ^TAM subset was approximately 42% on Day 10 post-tumor inoculation, whereas the proportion in the MHC class II^hi ^TAM subset was only around 18% (Figure 4C). We next examined changes in the levels of the two subsets within tumor tissues during tumor progression. The proportion of MHC class II^hi ^TAMs was higher than that of MHC class II^low ^TAMs during the early stages of tumor development (Figure 4D). However, both the proportion and number of MHC class II^low ^TAMs markedly increased as the tumor progressed (Figure 4E), indicating that infiltrating MΦ preferentially differentiate into MHC class II^low ^TAMs as tumors continue to grow.

To determine the effects of Cl_2_MDP-liposomes on the two TAM subsets, we examined expression of MHC class II in both untreated and TAM-depleted tumors using immunohistochemistry and flow cytometry. As shown in Figure 4F, there were differences in the expression of MHC class II between untreated and TAM-depleted tumors. Although the total number of TAMs expressing MHC class II decreased after treatment with Cl_2_MDP-liposomes, the number of MHC class II^low ^TAMs in the treated tumors was significantly lower than in untreated tumors. In contrast, the number of MHC class II^hi ^TAMs was not significantly affected by treatment with Cl_2_MDP-liposomes (Figure 4G and 4H). Taken in the context of the data presented in Figure 3A, these results suggest that after depleting the majority of TAMs, the remaining MΦ undergo transition from MHC class II^hi ^to MHC class II^low ^at an accelerated rate, resulting in delayed tumor growth.

Taken together, these data demonstrate that two TAM subsets exist within the tumor tissues and that transition between the two sub-populations is closely related to tumor progression, during which the predominant MHC class II^hi ^subset may shift to an MHC class II^low ^subset.

### MHC class II^low ^TAMs promote tumor growth and MHC class II^hi ^TAMs promote tumor inhibition

**Figure 5:**
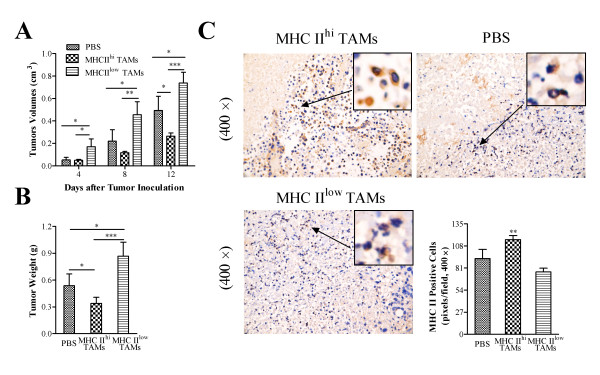
**Adoptive transfers of TAM subsets**. For the adoptive transfer experiments, 2 × 10^6 ^sorted F4/80^+^MHC II^hi ^and F4/80^+^MHC II^low ^cells derived from tumors on Day 21 after inoculation (*n *= 20) were injected into the tumor tissues of other mice 2 days after tumor inoculation (*n *= 4). PBS was used as the control. (A) Tumor size. (B) Tumor weight. (C) MHC class II expression in the differently treated tumors was examined by immunostaining and counting (*n *= 4) (× 400 magnification). The bars represent the mean ± SD; *p < 0.05, **p < 0.01, ***p < 0.001 (unpaired Student's *t*-test).

To further characterize the effects of the two heterogeneous TAM subsets on tumor progression, the MHC class II^hi ^and MHC class II^low ^cells were sorted and adoptively transferred to tumor-bearing mice 2 days after tumor inoculation. As expected, compared with the control group (PBS), tumor progression was significantly induced in mice treated with MHC class II^low ^TAMs, but markedly inhibited in mice treated with MHC class II^hi ^TAMs (Figure 5A). These results were verified by measuring tumor weight and size (Figure 5B). We then analyzed the expression of MHC class II molecules within the tumor tissues using immunohistochemistry. As shown in Figure 5C, the number of MHC class II-expressing cells in the tumors from the control mice and from mice treated with MHC class II^low ^was significantly lower than that in the tumors from mice treated with MHC class II^hi ^TAMs. These results suggest that tumor progression is positively correlated with the number of infiltrating MHC class II^low ^TAMs, but negatively correlated with the number of infiltrating MHC class II^hi ^TAMs.

### MHC class II^low ^TAMs inhibit T cell activation and promote invasion of tumor cells by secreting immunosuppressive factors

**Figure 6:**
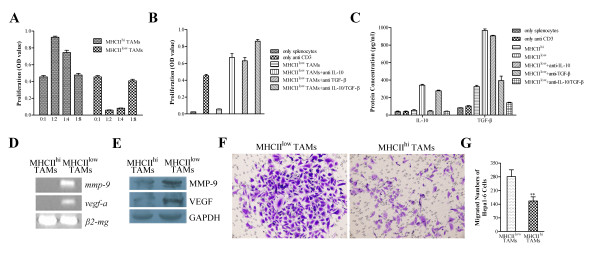
**MHC class II^low/hi ^TAM subsets modulate T cell proliferation through IL-10/TGF-β production and invasion and migration of tumor cells through MMP-9 and VEGF production**. (A) Suppression or promotion of anti-CD3-induced T cell proliferation by different TAM subsets (*n *= 3). (B) The suppression of T cell polyclonal activation was reversed by neutralizing IL-10 and TGF-β (*n *= 3). (C) IL-10 and TGF-β levels after co-culture of TAMs and splenocytes (*n *= 3). (D) RT-PCR analysis of *mmp-9 *and *vegf *expression. (E) Western blot analysis of MMP-9 and VEGF expression. (F) Representative images showing tumor cell migration taken 24 h after co-culture of Hepa1-6 cells and TAMs (× 400 magnification). Images shown are representative of three independent experiments. (G) Number of migrated tumor cells. The bars represent the mean ± SD; **p < 0.01 (unpaired Student's *t*-test).

Since T cell-mediated cellular immunity plays a critical role in the regulation of tumor progression, we examined the contribution made by each of the TAM subsets to the activation of T cells. A T cell polyclonal activation assay was performed using the TAM subsets. Sorted MHC class II^hi ^and MHC class II^low ^TAMs were co-cultured with allogeneic splenocytes in the presence of an anti-CD3 antibody. T cell proliferation was measured using the BrdU assay. MHC class II^hi ^TAMs promoted anti-CD3-induced T cell proliferation in a dose-dependent manner, whereas MHC class II^low ^TAMs suppressed anti-CD3-induced T cell proliferation. The increasing number of MHC class II^low ^TAMs correlated with a decrease in T cell proliferation (Figure 6A). MHC class II^low ^TAMs express high levels of IL-10 and TGF-β, which may contribute to T cell inactivation. This was examined using antibodies to block IL-10 and TGF-β. As shown in Figure 6B, T cell activation was significantly inhibited in the absence of the blocking antibodies. This was reversed by neutralizing IL-10 and TGF-β. These results were further verified by measuring the levels of IL-10 and TGF-β in the supernatants of TAMs co-cultured with splenocytes (Figure 6C). The results suggest that MHC class II^low ^TAMs contribute to tumor progression via IL-10/TGF-β-mediated suppression of T cell activation.

We also examined the expression of MMP-9 and VEGF in both subsets using RT-PCR and western blotting. The expression of MMP-9 and VEGF in MHC class II^hi ^TAMs was lower than that in MHC class II^low ^TAMs (Figure 6D and 6E). To assess the effects of the different TAM subsets on the invasiveness and metastasis of mouse hepatoma cells, an invasion assay was performed using HCC cells. The invasiveness of cancer cells was markedly enhanced by MHC class II^low ^TAMs (*p *< 0.01; Figure 6F and 6G). This result suggests that MHC class II^low ^TAMs promote cell invasion to a greater extent than MHC class II^hi ^TAMs.

## Discussion

This study showed that partial depletion of MΦ by Cl_2_MDP-liposomes efficiently modulates the tumor microenvironment and tumor progression in the Hepa1-6 cell-transplanted tumor model. Furthermore, heterogeneous TAM populations were found to coexist in the tumor microenvironment. MHC class II^hi ^TAMs appeared during the early phases of tumor development and showed tumor suppressive activity. In contrast, MHC class II^low ^TAMs became more dominant as the tumors progressed, and were alternatively (rather than classically) activated and promoted tumor growth.

Cl_2_MDP is a bisphosphonate developed to treat osteolytic bone disease [25]. The free drug does not easily enter cells and has an extremely short half-life in the circulation and body fluids. However, it is ingested specifically by MΦ and has a longer half-life when encapsulated in liposomes [21,22]. MΦ are irreversibly damaged owing to intracellular accumulation of the drug and undergo apoptosis because of the formation of toxic ATP analogues [21,22,25]. This strategy efficiently reduces the number of MΦ *in vivo *and has been used successfully to investigate the role of MΦ in animals [7,23,24,26,27]. The results of the present study show that treatment with Cl_2_MDP-liposomes efficiently and specifically reduces the number of TAMs in a murine transplanted hepatoma model. Although tumor regression following TAM depletion has been reported in a human hepatoma xenograft nude mouse model treated with sorafenib, treatment with Cl_2_MDP-liposomes alone has no inhibitory effect on tumor growth in tumor-bearing nude mice [27]. This disparity can be explained by the absence of T cell-mediated cellular immune responses in nude mice. Another possible explanation is that the effects of Cl_2_MDP-liposome treatment are limited in nude mice. That said, the role of MΦ in tumor-bearing mice and rats is still extensively studied using Cl_2_MDP-liposomes.

The choice of administration route used for liposome treatment enables some degree of selectivity with respect to the MΦ populations targeted [28]. Injection of Cl_2_MDP-liposomes via the i.v. route depletes MΦ in liver, spleen, bone, and circulation, whereas s.c. injection only affects MΦ in the lymph nodes [28]. A recent study shows that intratumoral (i.t.) injection of Cl_2_MDP-liposomes into tumor-bearing mice significantly delays tumor growth [29]. Although the total number of MΦ affected by i.p. injection is higher than that affected by other routes (e.g. the i.v. route), it necessitates the use of a relatively large volume of Cl_2_MDP-liposomes [28]. Thus, in the present study, we injected Cl_2_MDP-liposomes via the i.v. route for orthotopically implanted tumors, and via both i.v. and i.t. routes in the s.c.-implanted tumor model, to maximally reduce the number of TAMs infiltrating the tumors and to minimize any possible toxic side effects mediated by the Cl_2_MDP-liposomes. We did not examine the effect of i.v. or i.t. injection of Cl_2_MDP-liposomes on s.c.-transplanted tumors, as the combination of i.v. injection plus i.t. administration was thought to be the effective procedure for depleting TAMs in our models. However, the choice of injection route needs to be validated by additional investigations.

Unlike lymphocytes, MΦ do not express specific markers, although F4/80, CD68, and CD11b are commonly used to label these cells. A rat monoclonal anti-MΦ antibody [RM0029-11H3] was developed against isolated mouse MΦ and was used to label MΦ in some studies [30,31]. A recent study shows that MΦ can be classified into CD11b^+^Ly6C^hi ^and CD11b^+^Ly6C^low ^populations based on their expression of CD11b and Ly6C [19]. Differences in the expression levels of these biomarkers can be used to examine MΦ subpopulations. In the present study, histological analyses showed that TAMs could be exclusively labeled using antibodies to F4/80, CD68, and with the rat monoclonal anti-MΦ antibody [RM0029-11H3], although there were quantitative differences in the expression levels of these biomarkers.

MΦ within tumors are polarized toward the M2 phenotype by tumor-derived factors, which cause them to promote tumor progression [3,4,14,32-34]. IL-10 and TGF-β play an important role in promoting tumor growth. IL-10 is likely to induce TAM-derived CCL18, which is a chemoattractant for naive T cells, followed by T cell anergy via altered STAT1 expression [35-37]. The results of the present study showed that MHC class II^low ^TAMs expressed high levels of IL-10 and TGF-β. The role of these cytokines was verified, at least in part, by blocking cytokine-mediated T cell suppression *in vitro *using anti-IL-10 and anti-TGF-β mAbs. Interestingly, the levels of IL-10 and TGF-β, which are expressed by MHC class II^low ^TAMs in the TS/A tumor model, are low [19]. This may be explained by disparities in the experimental models; for example, the constitution of the tumor microenvironment may be different. There may also be a more complex mechanism that mediates suppression of T-cell polyclonal activation by MHC class II^low ^TAMs in the TS/A tumor model. However, MHC class II^low ^TAMs in the Hepa1-6 tumor model are more likely to mediate suppression of T-cell activation via production of IL-10 and TGF-β. Although the exact mechanisms underlying MHC class II^low ^TAM-mediated suppression of tumor growth *in vivo *are yet to be elucidated, our observations provide an insight into how TAMs subvert anti-tumor adaptive responses and redirect T cell activity to favor tumor growth. TAM-mediated T cell anergy may play an important role in tumor promotion. Our investigation also shows that MHC class II^low ^TAMs promote invasion of Hepa1-6 cells via the production of angiogenesis-associated molecules such as VEGF and MMP-9. This suggests that MHC class II^low ^TAM subset-mediated angiogenesis could be another important cause of runway tumor progression. Thus, the mechanisms underlying TAM subset-mediated tumor growth are intricate and need further study.

TAMs are a heterogeneous population that may have both inhibitory and stimulatory influences on malignant growth [38]. The transition between complex phenotypes depends on the tumor milieu [19,39]. For example, studies in patients with HCC reveal that two distinct populations (HLA-DR^high ^and HLA-DR^low ^IL-10^+^) of monocytes/MΦ exist within different regions of the tumor, which modulate T cell activation via different mechanisms [4,5]. Although the MHC class II^hi ^TAM and MHC class II^low ^TAM populations in the TS/A tumor model mediate tumor progression through different mechanisms, dynamic analysis of TAM subsets shows that there is a balance between these populations [19]. This is consistent with our observation that a shift from MHC class II^hi ^to MHC class II^low ^in the Hepa1-6 tumor model correlates with tumor progression. Infiltrating monocytes are more likely to differentiate into MHC class II^hi ^TAMs during the early phase of tumor development, whereas MHC class II^low ^TAMs become gradually more prominent as the tumors progress. However, this study also showed that MHC class II^low ^TAMs promoted tumor progression, whereas MHC class II^hi ^TAMs had a suppressive effect on tumor growth. Further investigation into the effects of the tumor microenvironment on the balance between TAM subsets may help to explain this discrepancy.

## Conclusions

This study shows that TAM depletion causes tumor regression in a murine hepatoma xenograft mouse model. Heterogeneous TAM subsets co-exist within the tumor microenvironment, and there is a transition from MHC class II^hi ^to MHC class II^low ^TAMs that correlates with tumor progression. The MHC class II^hi ^TAM population appears during the early phase of tumor development and contributes to tumor suppression, whereas the MHC class II^low ^population becomes dominant as the tumor progresses. MHC class II^low ^TAMs are alternatively activated and promote tumor growth. Therefore, targeting the transition of MΦ may be a novel strategy for drug development and immunotherapy.

## Methods

### Reagents

The following reagents were used in the study: Rat anti-mouse F4/80 mAb (AbD; Serotec, Kidlington, UK), a rat anti-MΦ mAb [RM0029-11H3] (a rat monoclonal antibody against isolated mouse MΦ; Abcam, Cambridge, MA, USA), rat anti-mouse MHC class II (I-A^b,d,q^/I-E^d,k^) mAb (BioLegend, San Diego, CA, USA), and rat anti-mouse-IL-10/TGF-β and rabbit-anti-mouse-CD31/VEGF/MMP-9 mAb (Santa Cruz Biotechnology, Santa Cruz, CA, USA). The IL-10 and TGF-β enzyme-linked immunosorbent assay (ELISA) kits were purchased from Bender Medsystems (GmbH, Vienna, Austria). All fluorescently-labeled mAbs and isotype control mAbs were obtained from eBioscience (San Diego, CA, USA). Clodronate (dichloromethylene diphosphonate, Cl_2_MDP) was a generous gift from Roche Diagnostics (GmbH, Mannheim, Germany). All other reagents were obtained from Sigma. (St Louis, MO, USA) unless otherwise indicated.

### Animal care

Female C57BL/6 and BALB/c mice (6-8 weeks old) were obtained from Beijing Vital River Experimental Animals, Co. Ltd. All animal experiments were approved by the Laboratory Animal Committee of Guangzhou Institutes of Biomedicine and Health, Chinese Academy of Science.

To investigate the effects of Cl_2_MDP-liposomes on tumor growth, naïve mice were first injected i.v. with 200 μl of C1_2_MDP-liposome suspension 2 days prior to tumor inoculation. Peripheral blood (50 μl) was collected retro-orbitally 0, 24, and 48 h after treatment and immediately transferred to ethylenediaminetetraacetic acid (EDTA) -containing tubes (BD Bioscience). Whole blood cells were stained with APC-conjugated anti-CD11b and FITC-conjugated anti-F4/80 mAbs for 30 min in the dark on ice. The cells were then washed and the red blood cells lysed with FACS lysis buffer (BD Bioscience). The remaining white blood cells were washed again and resuspended in PBS containing 1% fetal calf serum (FCS). Flow cytometry data were acquired using a BD FACSAria and analyzed using FLOWJO software version 7.6.0 (Tree Star, Inc., Ashland, OR, USA). After tumor inoculation, mice were treated with Cl_2_MDP-liposomes via both i.v. (200 μl) and i.t. (50 μl) routes on Days 3, 8, 13, and 18. PBS-liposomes and saline were used as the controls. Tumor size was measured every 4 days using calipers and calculated using the formula: 0.52 × a × b^2 ^(a: long diameter of the tumor; b: short diameter of the tumor). After 20 days, tumor-bearing mice were sacrificed for further analysis.

For the orthotopic tumor model, 200 μl of Cl_2_MDP-liposomes were injected i.v. on Day 2 before surgery, and at 3, 8, and 13 days after surgery. After 15 days, mice were sacrificed and the tumors were removed and weighed.

### Preparation of Cl_2_MDP-liposomes

Cl_2_MDP-liposomes were prepared as described previously [21,22]. Briefly, a mixture of 8 mg of cholesterol and 86 mg of phosphatidylcholine was prepared in chloroform in a round-bottom flask. The thin film on the interior of the flask after low-vacuum evaporation at 37°C was dissolved in 10 ml of 0.7 M Cl_2_MDP solution and incubated for 2 h at room temperature under Argon gas protection, followed by 3 min of sonication and another 2 h of incubation at room temperature. The liposomes were washed five times by centrifugation to remove any free drugs. The final pellet was resuspended in 4 ml of sterile PBS. The control liposomes were prepared using PBS. Cl_2_MDP-liposomes and PBS-liposomes were stored at 4°C for 2 weeks.

### Cell culture and tumor models

The murine HCC cell line, Hepa1-6, and the murine MΦ cell line, RAW264.7, were cultured in DMEM supplemented with 10% FCS and 1% penicillin-streptomycin at 37°C. For the s.c. tumor model, 100 μl of serum-free RPMI 1640 containing 2 × 10^6 ^Hepa1-6 cells was injected s.c. into the right anterior axillary fossa of naïve mice. A palpable spherical mass emerged after 4 to 6 days.

An orthotopic hepatoma model was created by transplantation of a small tumor mass into the left liver lobe of naïve mice. Briefly, 2 × 10^6 ^Hepa1-6 cells was injected s.c. into the right anterior axillary fossa of naïve mice. The mice were sacrificed when the hepatoma grew progressively and the tumor diameter exceeded 10 mm. The tumors were removed and chopped into small pieces (about 1 mm^3^) using a scalpel and forceps. The small masses were surgically implanted under anesthesia (a small tumor mass/mouse) into one site in the left liver lobe of mice treated with Cl_2_MDP-liposomes, PBS-liposomes, or saline.

### Immunohistochemical analysis

Preparation of histological specimens (from tumors on Day 20 after treatment with Cl_2_MDP-liposomes or from tumors on Day 10 after adoptive transfer of sorted TAM populations) and immunostaining were carried out as described previously [35]. Antigen retrieval was performed under high pressure in 10 mM EDTA buffer (pH 8.0) before incubation with primary antibodies to F4/80, CD68, or rat monoclonal anti-MΦ [RM0029-11H3] or IL-10, TGF-β, VEGF, MMP-9, CD31 and MHC class II. Brightfield images were then captured and analyzed.

The number of infiltrating MΦ and the CD31-positive microvessel density were counted in 10 random fields at × 400 magnification (a 0.17 mm^2 ^field), and the results were expressed as the mean number per field ± SD (10 fields per tumor, 5 tumors per group).

### Isolation of TAMs and splenocytes

Single cells within the tumor tissue were isolated as described previously with some modifications [40]. Briefly, solid tumors were harvested from tumor-bearing mice on Days 7, 14, 21, and 28 after tumor inoculation or harvested under sterile conditions from tumor-bearing mice untreated or treated with Cl_2_MDP-liposomes. The tumors were chopped into small pieces using scissors and forceps before incubation with a mixture of enzymes dissolved in RPMI 1640 (400 U/ml collagenase type IV, 0.05 mg/ml collagenase type I, 0.025 mg/ml hyaluronidase, 0.01 mg/ml DNase I and 0.2 units/ml soybean trypsin inhibitor) for 30 min at 37°C. Cells were recovered by centrifugation and resuspended in PBS, containing 1% FBS. The debris and red blood cells were removed using the Ficoll density gradient method. F4/80^+^MHC II^hi ^and F4/80^+^MHC II^low ^cells were analyzed and sorted by flow cytometry. Briefly, cells were resuspended in PBS supplemented with 1% FCS, and 1 × 10^7 ^cells were incubated with 10 μg of FITC-conjugated F4/80 mAb and 0.2 μg of PE-conjugated MHC class II mAb for 30 min on ice. The cells were then washed with PBS supplemented with 1% FCS to remove unbound Abs. The F4/80^+^MHC II^hi ^and F4/80^+^MHC II^low ^cell populations were analyzed and sorted using a FACSAria flow cytometer. All data were analyzed using FLOWJO software version 7.6.0. The purity of the cell populations exceeded 90%.

Single cells were also prepared as described above on Day 10 after tumor inoculation. Briefly, 1 × 10^7 ^sorted cells were incubated with 2 μg of APC-conjugated F4/80 mAb and 0.2 μg of PE-conjugated MHC class II mAb for 30 min on ice. The cells were then washed with PBS supplemented with 1% FCS. After fixation, permeation, and washing, the cells were stained with 10 μg of FITC-conjugated IL-10 mAb. The unbound antibodies were removed by washing. The expression of IL-10 by F4/80^+^MHC II^hi ^and F4/80^+^MHC II^low ^cells was then analyzed.

Naïve BALB/c mice (6-8 weeks old) were sacrificed and the spleens were harvested using syringes and forceps and single cell suspensions obtained. Red blood cells were removed with ACK lysis buffer (0.15 M NH_4_Cl, 1 mM KHCO_3_, 0.1 mM EDTA, pH 7.3), and the remaining splenocytes were washed twice by centrifugation. The splenocytes were counted, diluted to the appropriate concentration in RPMI 1640 supplemented with 10% FCS and 1% penicillin-streptomycin for further investigation.

### Real-time quantitative reverse transcription PCR (qRT-PCR) and reverse transcription PCR (RT-PCR)

Total RNA from the sorted TAM populations was isolated using Trizol according to the manufacturer's instructions. qRT-PCR to examine the expression of *ym1, mgl1/2, fizz1, arg-1, msf, il-10, tgf-β, mmp-9, vegf, ptge2, il-1β, il-6, il-12*, and *inos *was performed using a One Step SYBR^® ^PrimeScript™ RT-PCR kit according to the manufacturer's instructions (Takara, Dalian, China). *β2-mG *was used as a the reference gene.

Two-step RT-PCR was performed to examine the expression of *mmp-9 *and *vegf *in the sorted TAM populations. cDNA was synthesized from 2 ug of total RNA using Moloney murine leukemia virus reverse transcriptase (Takara) after DNase treatment. The PCR conditions were as follows: 94°C for 5 min followed by 30 cycles at 94°C for 30 s, 60°C for 30 s, and 72°C for 40 s, with a final extension at 72°C for 7 min. The *β2-mG *gene was used as a reference. The PCR products were analyzed by gel electrophoresis.

### Western blot analysis

Total proteins were extracted from the sorted TAM populations and electrophoresed on 10% SDS polyacrylamide gels. The proteins were then transferred to polyvinylidene fluoride membranes (Millipore, MA, USA). Non-specific binding was blocked with 5% (wt/vol) nonfat milk in PBS-Tween-20 (0.1% vol/vol). The membranes were then incubated with primary antibodies against MMP-9 and VEGF at 4°C overnight and with the appropriate secondary antibodies at room temperature for 1 h. Immobilon Western HRP Chemiluminescent Substrate (Cell Signaling Technology, MA, USA) was used to detect the specific signals, and the band intensity was then visualized.

### Invasion and migration assays

Tumor cell invasion assays were performed using a Transwell apparatus (Corning, NY, USA) with an 8- μm-pore polycarbonate filter membrane. The upper sides of the membranes were pre-coated with Matrigel matrix (BD, NJ, USA) according to the manufacturer's instructions. Sorted TAMs (2 × 10^5^) were pre-seeded in the lower chamber. The upper chamber was seeded with 1 × 10^4 ^Hepa1-6 cells and inserted into the lower chamber. After incubation for 24 h, the cells on the interior of the upper chamber were removed by swabbing with cotton swabs. The polycarbonate membranes were fixed in methanol for 20 min and stained with 0.1% crystal violet for 15 min. The number of cells migrating to the underside of the membranes was counted in five randomly selected fields under a microscope. For the migration assay, the same procedure was used as described above but without addition of Matrigel to the top of the upper chamber membrane.

### T cell suppression assays

For the T cell suppression assays, 1 × 10^5 ^to 2.5 × 10^4 ^sorted TAMs were co-cultured with 2 × 10^5 ^naive splenocytes (1:2-1:8) and 1 μg/ml anti-CD3. To neutralize IL-10 and TGF-β, 500 ng/ml IL-10 and/or TGF-β mAb were added to the wells containing MHC class II^low ^TAMs, followed by BrdU staining 24 h later. T cell proliferation was assessed using a commercial ELISA kit (Roche Diagnostics, GmbH, Mannheim, Germany), according to the manufacturer's instructions.

### ELISA

The co-culture supernatants were collected and stored at -80°C until used. IL-10 and TGF-β levels were measured using commercially available ELISA kits (Bender Medsystems) according to the manufacturer's instructions.

### Adoptive transfer of distinct TAM subsets

For the adoptive transfer assays, 2 × 10^6 ^sorted F4/80^+^MHC II^hi ^and F4/80^+^MHC II^low ^cells were injected, into the tumors of mice 2 days after inoculation of Hepa1-6 cells. PBS was used as the control. Tumor size was measured every 4 days. The mice were sacrificed 10 days after adoptive transfer and the tumors dissected out for further analysis.

### Statistical analysis

Statistical significance was determined using the Student's *t *test and GraphPad Prism software. Data were expressed as the mean ± SD. A *p *value < 0.05 was considered significant.

## Authors' contributions

BW, LQ, JW, and XC designed the project, analyzed the data and wrote the manuscript. BW performed all the experiments. QL and SZ participated in the animal experiments. All authors read and approved the final manuscript.
